# Evaluation of the Therapeutic Potential of Histone Deacetylase 6 Inhibitors for Primary and Metastatic Uveal Melanoma

**DOI:** 10.3390/ijms23169378

**Published:** 2022-08-19

**Authors:** Husvinee Sundaramurthi, Zoltán Giricz, Breandán N. Kennedy

**Affiliations:** 1 UCD Conway Institute, University College Dublin, D04 V1W8 Dublin, Ireland; 2 UCD School of Biomolecular and Biomedical Science, University College Dublin, D04 V1W8 Dublin, Ireland; 3 Systems Biology Ireland, University College Dublin, D04 V1W8 Dublin, Ireland; 4 UCD School of Medicine, University College Dublin, D04 V1W8 Dublin, Ireland; 5 Pharmahungary Group, 6720 Szeged, Hungary; 6 Department of Pharmacology and Pharmacotherapy, Semmelweis University, 1085 Budapest, Hungary

**Keywords:** primary and metastatic uveal melanoma, HDAC6 inhibitors, HDAC isozymes, extracellular vesicles, combinatorial therapies

## Abstract

Patients diagnosed with metastatic uveal melanoma (MUM) have a poor survival prognosis. Unfortunately for this rare disease, there is no known cure and suitable therapeutic options are limited. HDAC6 inhibitors (HDAC6i) are currently in clinical trials for other cancers and show potential beneficial effects against tumor cell survival in vitro and in vivo. In MUM cells, HDAC6i show an anti-proliferative effect in vitro and in preclinical xenograft models. The use of HDAC6 inhibitors as a treatment option for MUM should be explored further. Therefore, this review discusses (1) what is known about HDAC6i in MUM and (2) whether HDAC6 inhibitors offer a potential therapeutic option for MUM.

## 1. Introduction

Uveal melanoma (UM) is a poor prognosis cancer with no cure and limited treatment options [1,2,3]. Originating in the eye, UM is the most common form of adult ocular cancer [4]. Metastatic UM (MUM) is difficult to treat. A recent meta-analysis estimated a 60% relative survival rate of 15 to 20 years for patients after initial UM diagnosis [5]. Approximately 50% of UM patients progress to MUM, with only 15% of patients reported to survive beyond one-year post diagnosis [1,4,6]. In a meta-analysis (1 January 1980 to 29 March 2017) from a combined 2494 MUM patients, the median overall survival probability was 1.07 years across all forms of treatment [7]. Common treatment strategies for MUM include chemotherapeutic agents, immunotherapy, site-directed (e.g., chemoembolization, immunoembolization) therapy and surgical tumor resection [1,2,8,9]. Additional targeted and combinatorial pharmacological-based treatment approaches such as receptor tyrosine kinases, MAPK pathway inhibitors, checkpoint inhibitors and histone deacetylase inhibitors have entered clinical trials [9,10,11,12].

A milestone in UM was achieved in January 2022 with FDA approval of Tebentafusp-tebn (KIMMTRAK^®^), an immunotherapy, for treatment of HLA-A*02:01-positive adult patients with unresectable or metastatic UM [13]. Very recently, Tebentafusp-tebn received EMA approval for use in the EU [14,15]. A phase III clinical trial (ClinicalTrials.gov Identifier: NCT03070392/EudraCT registration number: 2015-003153-18) reported that MUM patients (252) treated with Tebentafusp had improved one-year overall survival (OS) and six-months progression-free survival (PFS; 73% and 31%, respectively), on average, compared to patients (126) treated with control treatment (59% and 19%, respectively) options [13,16]. Overall it was reported that the patient cohort treated with Tebentafusp achieved 21.7 months median OS compared to 16 months (hazard ratio (HR) for death = 0.51; 95% CI 0.37 to 0.71; *p* < 0.001) in the control cohort; median PFS was 3.3 months in Tebentafusp treatment group compared to 2.9 months (HR for disease progression or death = 0.73; 95% CI, 0.58 to 0.94; *p* = 0.01) in the control group [13]. However, at present this drug is only approved for use in a sub-cohort of patients, making it essential that more therapies and therapeutic targets are discovered for the treatment of a wider cohort of patients. Additionally, in an article published by Olivier and Prasad (2022), the authors put forth three key concerns regarding this study; (1) limited treatment options offered to control patient cohort in trial, (2) only 43% of the trial patients received treatment for disease progression post trial, and (3) interestingly, the observed median OS is *“far larger than, and disproportionate to the PFS benefit in terms of hazard ratios”* and that mathematically, Tebentafusp *“is an outlier across trials in melanoma”*, suggesting that further investigation is required for Tebentafusp [17].

In recent decades, histone deacetylase inhibitors (HDACi) were investigated as anti-cancer agents, with some receiving FDA/EMA approval for T-cell lymphoma and multiple myeloma [18,19,20]. However, pan-HDACi are often skeptically considered due to histone deacetylase (HDAC) function in epigenetics, suggesting off-target effects and undesirable side-effects associated with the pleiotropic inhibition of HDAC isozymes. The true therapeutic potential of pan-HDACi in oncology remains to be determined. Several reviews have been published on HDACs and HDACi in cancer, and Moschos et al. eloquently summarized the potential of broad HDACi as anti-UM agents [21].

Selective histone deacetylase 6 inhibitors (HDAC6i) are small molecule drugs with higher affinity for HDAC6 inhibition than other HDAC isozymes or other targets. HDAC6i are currently in clinical trials for various maladies, including cancer [22,23]. Histone deacetylase 6 (HDAC6), classified as a Class IIb enzyme is different to other HDACs. HDAC6 primarily resides in the cytoplasm and deacetylates cytosolic proteins, although it has the ability to shuttle between the nucleus and cytoplasm [24,25]. HDAC6 is a Zn^2+^-dependent deacetylase composed of two deacetylases catalytic domains, dynein motor binding domain and ubiquitin-binding zinc-finger functional domains [26]. HDAC6 functions in cancer-related processes such as tumorigenesis, angiogenesis, metastasis, the aggresome–autophagy pathway and inflammation as detailed in previous review articles [22,23,27,28]. With its multifaceted roles, HDAC6 is an obvious therapeutic target that needs to be investigated thoroughly as a cancer treatment option. This review explores the possibility and limitations of HDAC6 inhibitors compared to pan-HDACi as novel treatment options for primary and metastatic UM. 

## 2. HDACi Clinical Trials for Uveal Melanoma and/or Metastatic Uveal Melanoma

A search in the International Clinical Trials Registry Platform (ICTRP) returned seven registered clinical trial studies evaluating the efficacy of pan-HDACi (stand-alone or in combination with chemotherapeutic drugs) in UM and MUM patients, with one study completed and data published (Table 1). 

Most recently, primary study results from an ongoing Phase II trial (ClinicalTrials.gov Identifier: NCT02697630/EudraCT registration number: 2016–002114-50) exploring the efficacy of an Entinostat (selective Class I HDACi) and Pembrolizumab (PD-1 inhibitor) combination treatment in MUM patients. It reported that 28% (8 out of 29 patients) showed a partial response or stable disease at 18 weeks defined as per RECIST v1.1 criteria. The median OS was 13.4 months with 59% one-year OS; and the median PFS was 2.1 months with a 17% one-year PFS, reported [29,30]. Another Phase II trial (ClinicalTrials.gov Identifier: NCT03765229) investigating the efficacy of combinatorial Entinostat and Pembrolizumab therapy is currently recruiting participants with non-inflamed, unresectable advanced (stage III/IV) melanoma, inclusive of patients with tumors originating from ocular melanoma. A completed Phase II (ClinicalTrials.gov Identifier: NCT00121225) study investigating the efficacy of Vorinostat (pan-HDACi) in metastatic melanoma or unresectable melanoma (inclusive of cutaneous and ocular origin) reported that 16 (5 primary UM patients) patients out of 32 had stable disease, with a reported median PFS of 5 months, though the primary end point (as defined by RECIST v1.0 criteria) was not met in this study [31]. 

Regardless, the authors recommended that Vorinostat should be examined in combination with other agents for advanced melanoma as approximately 50% of patients did show a positive response in the form of stable disease or partial response to treatment. In an ongoing Phase II trial (ClinicalTrials.gov Identifier: NCT01587352), the efficacy of Vorinostat in MUM patients is being investigated. A proof-of-concept study, exploring the capability of Vorinostat to transform Class 2 UM cells into normal melanocytes (ClinicalTrials.gov Identifier: NCT03022565), was withdrawn by the investigator. In an independent study, the ability of Valproic acid (pan-HDACi) to prevent tumor growth in patients diagnosed with high-risk UM is being assessed in a Phase II clinical trial (ClinicalTrials.gov Identifier: NCT02068586). A Phase II trial (ClinicalTrials.gov Identifier: NCT05170334) was registered in December 2021, and is actively recruiting patients, to investigate whether combinatorial treatment of Binimetinib (selective inhibitor of MEK) and Belinostat (pan-HDACi) can inhibit or reduce tumor growth in MUM patients. These studies provide evidence that HDACi have attained clinical relevance and use in the treatment of UM/MUM is being thoroughly investigated for clinical approval. 

## 3. Selective HDAC6i as a Therapeutic Option for Uveal Melanoma

Interestingly, there are presently no registered clinical trials involving selective HDAC6i in UM/MUM, even though there are clinical trials ongoing for relapsed or refractory multiple myeloma, lymphoma, non-small cell lung cancer, metastatic breast cancer and solid tumor (Table 2) [23,32,33,34]. Likewise, to date, few studies have assessed the efficacy of selective HDAC6i in UM or MUM cells in vitro or in vivo. However, evidence in vitro and in pre-clinical UM models suggests inhibiting HDAC6 may offer therapeutic benefit. Nencetti et al. reported that novel compound VS13, with increased HDAC6 selectivity, significantly reduced 92.1 and Mel270 (primary) UM cell viability (up to a 100% reduction) in a dose-dependent manner [35]. Additionally, ACY-1215, a selective HDAC6i, induced a dose-dependent, significant reduction in UM and MUM cell survival by up to 99.99% in vitro and significantly decreased UM cell fluorescence by 65% in zebrafish xenografts with MUM (OMM2.5) cells in vivo [36]. 

## 4. Involvement of HDAC6 in Tumor Growth, Survival and Progression

**Figure 1:**
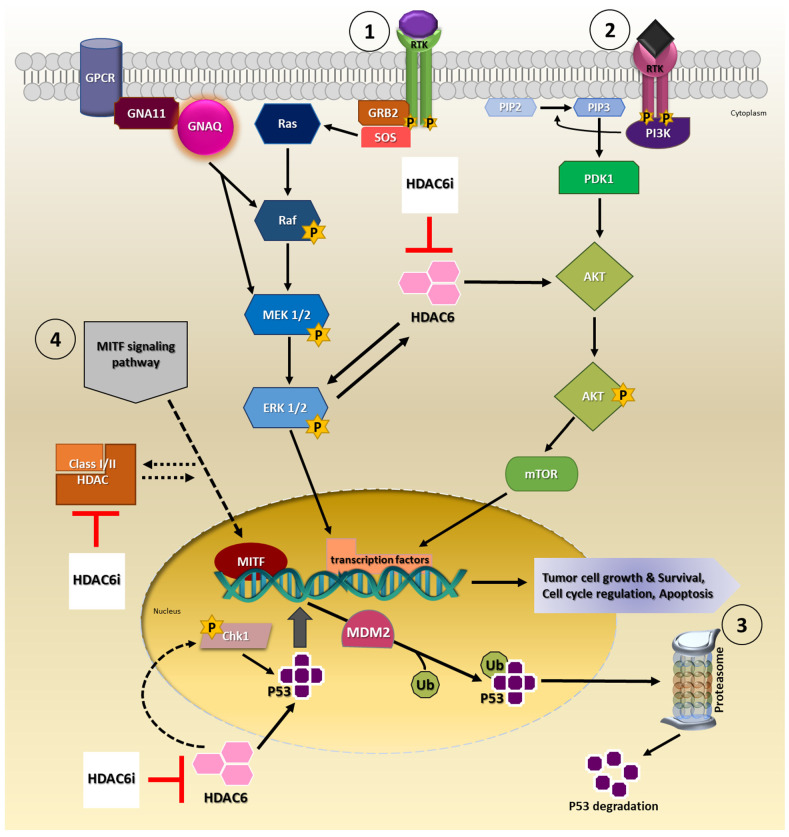
Involvement of HDAC6 in cancer signaling pathways. Proposed model for HDAC6i mechanism of action by targeting either ① MAPK/ERK, ② PI3K/AKT, ③ P53 and/or ④ MITF signaling pathway(s). Consequently, targeting these pathways inhibits biological processes that promotes cell survival and proliferation in the cancer cell.

Available evidence indicates HDAC6 is capable of modulating tumor growth, development, survival and progression via signaling pathways such as mitogen-activated protein kinases/extracellular-signal-regulated kinase (MAPK/ERK), phosphatidylinositol 3-kinase (PI3K/AKT) and p53 signaling cascade in cancers (Figure 1) [23,37,38,39,40,41,42]. Of these signaling pathways, MAPK/ERK and PI3K/AKT signaling, in particular, are of importance as they are implicated in UM disease pathomechanisms [43]. In the majority of UM tumors, MAPK/ERK signaling pathway is constitutively activated, which is partly attributed to mutations in genes, guanine nucleotide-binding protein G(q) subunit alpha (*GNAQ*) and guanine nucleotide-binding protein G(q) subunit alpha-11 (*GNA11*), upstream of this signaling cascade [44,45]. Uveal melanoma cells treated with a MEK and PI3K inhibitor combination inhibited cell proliferation and induced apoptosis [46]. Moreover, several other studies explored MEK and PI3K/AKT pathway inhibitors as a therapeutic option for UM; however, the therapeutic efficacy has been contentious [43,47,48,49,50]. 

Studies show modulation of HDAC6 in HEK293T cells, either by siRNA mediated knock-down or ACY-1215 pharmacological inhibition, led to increased phospho-ERK levels and ERK1/2 activation, respectively, hence regulating ERK signaling [51,52]. In LNCaP prostate cancer cells, treatment with Panobinostat inhibited HDAC6 activity and triggered ERK activation, which consequently resulted in the arrest of cell cycle [51]. In colorectal cancer cells, HCT116 and HT29, knock-down of HDAC6, blocked cell proliferation, migration and invasion in part via the MAPK/ERK signaling pathway [53]. A reduction in the expression levels of phospho-MEK, phospho-ERK and phospho-AKT was demonstrated in these cells. Peng et al. (2017), reported that inhibition of cell proliferation and survival with ACY-1215 and/or Vemurafenib in A375 melanoma cells was partly mediated through the inhibition of ERK activation [54]. Similarly, it was observed that ACY-1215 treatment of esophageal squamous cell carcinoma cells, EC109 and TE-1, led to a decrease in phospho-ERK1/2, phospho-AKT levels and inhibited cell proliferation and survival [55]. 

The PI3K/AKT signaling pathway is constitutively activated in UM which facilitates tumorigenesis processes such as cell survival, inhibition of apoptosis and angiogenesis [56]. In ACY-1215 treated cholangiocarcinoma cells, cell proliferation was blocked, and apoptosis was triggered via the PI3K/AKT pathway [57]. Kaliszczak et al. (2016) demonstrated that cotreatment of HCT116 with HDAC6i and pan-AKT inhibitor/dual PI3K/mTOR inhibitor enhanced anti-tumor effects both in vitro and in vivo [58]. Other studies reveal that dual inhibition with HDACi and/or PI3K/AKT/mTOR inhibitor effectively reduced tumor cell proliferation in cancer cells, e.g., prostate cancer, multiple myeloma, relapsed or refractory diffuse large B-cell lymphoma, neuroblastoma, hematologic tumor(s), hepatocarcinoma and breast cancer cells [59,60,61,62,63]. 

Another significant pathway involved in UM disease pathogenesis may be attributed to the p53 signaling pathway. Mutation(s) in *TP53* is rare in UM tumors; however, its activity is commonly disrupted as a consequence of mutations and dysregulation of other key factors in this pathway [64,65]. Cao et al. demonstrated in vitro and in vivo, that ACY-1215 increased the transcriptional activity of p53, which consequently led to cell cycle arrest and apoptosis in triple-negative breast cancer cells [66]. In colorectal cancer cells, HDAC6 inhibition upregulated p53 levels and increased acetylated p53 levels, and consequently increasing apoptosis [67]. In another study, the authors proposed that in ovarian cancer cells, HDAC6 inhibition in combination with Paclitaxel activated p53 and induced apoptosis [40]. Recently, it was reported that ACY-1215 inhibited cell proliferation through the promotion of apoptosis through mitotic catastrophe in a p53-dependent manner by repressing p-Chk1 activity, in head and neck carcinoma cells [68]. Promisingly, a Class III specific HDAC inhibitor, Tenovin-6, prevented tumor cell growth and induced apoptosis by increasing p53 expression in uveal melanoma tumor and cancer stem cells [69]. 

Interestingly, an additional pathway receiving renewed attention is the microphthalmia-associated transcription factor (MITF) signaling pathway in UM. MITF signaling is of particular interest in the context of UM as mutations in this transcription factor and its interacting partners are associated with oncogenic functions and disease pathogenesis in cutaneous melanoma [70,71,72,73]. MITF is widely known as the master regulator of melanogenesis and melanocyte differentiation, in addition to regulating various cellular processes [74]. Comparably, in UM, MITF seems to have an intricate role balancing between UM tumor growth and tumor suppression activities [75]. Most recently, Phelps et al. presented that MITF acts as a bona fide tumor suppressor in UM [76]. The authors report using zebrafish models that loss of *mitfa* resulted in growth of UM tumors. In our study, we show a potential link between ACY-1215 inhibition and regulation of MITF in UM cells in vitro [36]. Contrarily, ACY-1215 treatment of MUM cells significantly downregulated MITF and MITF signaling pathway associated protein(s) expression levels, which probably contributed to the observed anti-tumor effects. Previously, Yokoyama et al. (2008) has also shown that pan-HDACi repressed M-MITF expression in clear cell sarcoma and melanoma cells [77]. Regardless, the exact relationship between HDACi/HDAC6i and MITF still remains to be determined.

As such these pathways offer additional novel putative targets that needs to be thoroughly investigated. Taken together, there is a strong possibility that using HDAC6i may offer therapeutic benefits mediated through the regulation of these highlighted pathways either as a monotherapy or as combinatorial therapy for UM, advanced UM and/or other solid tumors, which needs to be explored extensively. 

## 5. Immunomodulatory Effects of HDAC6 Inhibitors

The tumor microenvironment (TME) supports processes involved in tumorigenesis [78,79]. Composed of tumor cells, immune cells, stromal cells, signaling molecules, extracellular matrix and blood vessels, the microenvironment can ensure tumor cells are able to grow, survive, spread and even to gain resistance to therapy [79]. An increasing number of studies are focusing on understanding and identifying novel therapeutic targets within the tumor microenvironment for cancer treatment. In UM, tumors with chromosomal defects such as loss of one copy of chromosome 3 or gain of chromosome 8q, present with increased levels of inflammatory mediators and immune cells leading to a tumor-promoting inflammatory TME [80]. Differing from other cancers, increased amounts of tumor infiltrating lymphocytes (TILs) and tumor associated macrophages (TAMs) are correlated to poor prognosis and high metastasis risk in UM [81,82]. A delicate balance is required to ensure the prevention of UM cells from evading immune surveillance. 

HDACi are capable of immunomodulation in cancer [38,83,84,85,86]. Inhibition of Class I HDACs by HDACi or in combination with the immunomodulatory drug Lenalidomide, resulted in the downregulation of cellular Myc proto-oncogene protein (c-MYC) and increased cytotoxicity in multiple myeloma cells [87]. Additionally, ACY-1215 treatment, alone or combined with Lenalidomide, significantly reduced c-MYC, IKAROS family zinc finger (IKZF)1/IKZF3 and interferon regulatory factor 4 (IRF4) expression levels triggering immune system activation, which was postulated to be involved in the anti-tumor cell survival effects. The HDAC6 inhibitor A452 in combination with Lenalidomide or Pomalidomide, displayed significantly increased synergistic anti-proliferative effects which was attributed to the augmented reduction of c-MYC, IKZF1/IKZF3 and IRF4 expression in multiple myeloma cells [88]. In a preclinical mouse model of non-small cell lung cancer (NSCLC), ACY-1215 treatment increased the expression of MHC Class II molecules, CD86 and CD96 co-stimulatory molecules, suggesting that ACY-1215 may play a role in T-cell activation and antigen presentation, consequently promoting anti-tumor immunity [89]. Another study described that the HDAC6 inhibitor ACY-241 alone, and when combined with Oxaliplatin (chemotherapy drug), promoted T cell functions, thereby increasing the immunogenicity of tumor cells, in an NSCLC mouse model [90]. Inhibition of HDAC6 in melanoma cells resulted in the increased expression of MHC class I and presentation of tumor-related antigens [91]. Knox et al. demonstrated that treatment of murine melanoma model with the HDAC6i Nexturastat A, combined with the anti-PD1 antibody, increased tumor infiltration of CD8^+^ and natural killer cells and reduced the level of pro-tumorigenic M2 macrophages [92]. Combining HDAC6i with immunomodulatory agents can improve therapeutic efficacy [93]. Taken together, we consider that the tumor microenvironment in UM, especially MUM, and the role and mechanism of action of HDAC6i in the context of UM/MUM, should be thoroughly investigated.

## 6. Is HDAC6 Expression Disease-Relevant in Uveal Melanoma Tissues?

Perversely, high or low HDAC6 expression levels are associated with disease pathogenesis in different cancers. For example, in colon cancer, patient-derived tumor samples express significantly increased HDAC6 protein levels and this correlated with poor overall survival prognosis [53]. Similarly, high mRNA and/or protein HDAC6 expression levels are noted in esophageal squamous cell cancer, pancreatic cancer and lung adenocarcinoma [94,95,96]. In contrast, immunohistochemistry (IHC) analysis revealed that in diffuse large B-cell lymphoma (DLBCL) and breast cancer, high HDAC6 expression correlated with better overall, progression-free and disease-free survival, respectively; and overall survival in high-grade serous ovarian cancer (HGSOC) [97,98,99]. However, Yano et al. reported that IHC analysis of advanced HGSOC patient samples, high HDAC6 expression was associated with poorer overall and progression free survival [100]. Contradicting data have been reported in liver and gastric cancers as well [101]. Nevertheless, these data highlight that correlations between HDAC6 expression levels and disease prognosis should be analyzed in UM/MUM. 

In the context of UM, HDAC6 is expressed in the nucleus and cytoplasm of UM tissue with variable staining intensity observed in the 16 patient samples analyzed by IHC [102]. In another study, with 64 UM samples, there was no correlation observable between HDAC6 mRNA expression levels and high-risk UM nor HLA Class I expression levels [103]. More recently, Levidou et al. reported that HDAC6 expression in 72 UM tissue samples correlated with a significantly higher mitotic index, which relates to poor cancer prognosis but a significant correlation to overall survival and disease-free survival was not found [104]. In apparent contrast, *HDAC6* transcript levels analyzed in the TCGA database reported low *HDAC6* expression levels correlated with significantly poorer overall and disease-specific survival [36,105].

It still needs to be determined whether HDAC6 expression has prognostic value in metastatic UM patient samples. Together, these data suggest that targeting HDAC6 in UM and/or MUM samples may be advantageous. An in-depth analysis of HDAC isozyme expression levels in a larger UM and MUM patient cohort is warranted to understand whether there is a potential involvement for HDAC6 in MUM disease pathogenesis and if there is a correlation to disease prognosis. 

## 7. Highly Effective Anti-Cancer Activity of HDAC6 Inhibitors Achieved at Non-HDAC6 Selective Concentrations

In UM, evaluation of HDAC6 as a therapeutic target is warranted as HDAC6 is expressed in UM tissue. Likewise, evaluation of HDAC6i is warranted in UM based on efficacy in other cancers and in pre-clinical UM models. However, caution is needed as selective HDAC6i can act non-selectively at higher concentrations (Table 3) [106,107]. When HDAC6 was knocked out in HAP1 cells, treatment with highly-selective HDAC6i (Tubathian A, Tubastatin A, Tubacin and Ricolinostat) at non-selective concentrations still significantly reduced cell confluency [106]. In another study, treatment with the selective HDAC6i, Ricolinostat or Citarinostat, still resulted in dose-dependent inhibition of cell survival in CRISPR-induced HDAC6 knock-out melanoma (A375 cells), colorectal (DLD1 cells), triple negative breast cancer (MDA-MB-231) and ovarian (TOV-21G cells) cell lines [107]. Higher, non-selective concentrations of Ricolinostat was also required to reduce cell proliferation and migration in high-grade serous ovarian cancer cells [99]. Ricolinostat also had highly efficacious anti-proliferative properties under non-selective HDAC6 inhibitory concentration in UM and MUM cell lines [36]. Ultimately, if HDAC6i are safe and effective, the off-target pharmacology is acceptable. 

Some opinions suggest that more clinical benefit from selective HDACi and/or pan-HDACi will be achieved in combinatorial therapy, rather than monotherapy, as evidenced by ongoing clinical trials [20]. A consensus may be drawn from these papers that, the beneficial anti-cancer effects of these drugs should not be written off due to its effects achieved at non-selective doses. Even if a small molecule acts on non-specific targets, if the therapeutic benefit outweighs the negative off-target effects and/or unwanted side-effects, they should still be explored as suitable therapeutics. Therefore, there is still plenty of evidence available to indicate that HDAC6i still offers a novel therapeutic for UM/MUM. 

One caveat surrounding development of HDACi or HDAC6i as clinical cancer therapeutics is poor in vivo pharmacokinetics. As previously comprehensively reviewed by McClure et al., many HDACi display in vivo half-lives of less than 4 h and even ones with longer half-lives display low peak serum concentrations due to dose-limiting toxicity [19]. For example, the pan-HDACi, suberoylanilide hydroxamic acid (SAHA), had a half-life of approximately 120 min administered orally or approximately 40 min by I.V. administration [108]. An oral formulation of MS-275 in patients had a half-life of up to 80 h but resulted in toxicity and side effects [109]. Several market-approved HDACi are hydroxyamic acids which present with diminished clinical efficacy in part due to poor pharmacokinetics. The hydroxamic acid (zinc binding group) pharmacophore may lead to non-selectivity as well as affecting pharmacokinetic properties [110]. To circumvent these issues, studies are focused on synthesizing novel HDACi and/or HDAC6i to present with increased affinity and/or selectivity resulting in increased efficacy and reduced toxicity [111]. Another promising solution in preclinical studies is HDACi designed as prodrugs, and HDAC6i prodrugs should be considered in UM [112]. For example, Schlimme et al. reported that a novel selective HDAC6 inhibitor with a carbamate derivative was capable of acting as a prodrug in vitro [113]. In another study, the selective HDAC6i Bufexamac was modified to a carbamate prodrug which presented with increased selectivity for HDAC6 inhibition and was effective against cell proliferation in HL60 cells [114]. Other strategies such as HDAC6 selective inhibitors, chimeric HDAC6 inhibitors and HDAC6 PROTACs are currently being explored, and these are discussed in detail by Xiao et al. in a recent review [115]. Collectively, these studies provide evidence that HDACi or selective HDAC6i with improved bioavailability in vivo may be developed.

## 8. Future Perspectives

### 8.1. Drug Synergism as a Strategic Approach to Treat MUM

With multiple clinical trials ongoing for different solid tumors and or hematological malignancies, there are more promising data obtained reflecting that HDAC6 inhibitors offer an increased beneficial effect when used in combination with other cancer therapeutics (Table 2). There are multiple papers highlighting the increased benefits of using HDACi in combinatorial strategies in various other cancers [21,28,116]. ACY-1215, a HDAC6i, is in preclinical and clinical trials with known chemotherapeutics. Going forward, synergistic drug effects with HDAC6i needs to be thoroughly assessed and investigated, as this may be the key to discovering highly efficacious therapeutic options for UM/MUM.

Current evidence for combinatorial therapy with pan-HDACi includes the treatment of MUM patients with Entinostat and Pembrolizumab that resulted in an improved median OS and PFS of 13.4 months (compared to 10 months—derived from historical data) and 2.1 months, respectively [29,30]. Faiao-Flores et al. showed that treatment of UM cells in combination with MEK-HDAC (Trametinib and Panobinostat) inhibitors increased the anti-proliferative and cell death effects in vitro; and significantly reduced subcutaneous and liver tumor growth in vivo in UM xenograft mice models, in comparison to single agent treatment [47]. UM cell viability is compromised with increased apoptosis when treated with either Tenovin-6 (Class III specific HDACi) and Vinblastine (conventional chemotherapeutic agent) with a reported combination index of <1; or JSL-1 and Vinblastine as evidenced by increased cleaved PARP levels [69,117]. In another study, co-treatment of UM cells with HDAC-CDK (Quisinostat and Flavopiridol) inhibitor significantly reduced (combination index calculated: 0.9>) cell proliferation in a dose-dependent manner [118]. 

In future, stratification of patients according to their genetic background may also offer an option for increased efficacy with treatment strategies. However, these studies are still in their infancy; therefore, an in-depth analysis is warranted to realize the complete benefit of HDACi/HDAC6i as a stand-alone or combinatorial therapeutic option for UM/MUM. The use of orthotopic human UM/MUM xenograft models to efficiently screen drug combinations in vivo further creates the opportunity to establish the best treatment plan that offers the most benefit to individuals [119]. 

### 8.2. Can Extracellular Vesicles Be Exploited as Therapeutic Options in UM/MUM?

Since extracellular vesicles (EVs) can be engineered to carry therapeutic agents (such as HDAC inhibitors) specifically to diseased tissues, one can hypothesize that EVs may be used as therapeutic tools in UM or MUM; however, this is a relatively new and untapped niche [120]. A complex artificial vesicular structure consisting of nanoparticles, mesoporous silica and macrophage-derived EV membranes was loaded with the HDAC inhibitor SAHA and observed to reduce lung cancer progression more efficiently than the nascent compound [121]. This effect was attributed to the increased homing of artificial vesicles due to their integrin α4β1 content originated from the macrophage EVs [121,122]. This approach may be harnessed in case of UM, in particular MUM with liver metastases, given that EVs are taken up by the liver rapidly and that promising results have been published with EVs in other hepatic carcinomas [123].

Although cancers modulate EV-related processes, the disease and therapeutic relevance of EVs in UM need further investigation. In a single, small-scale study, the amount of EVs in circulation was approximately five-fold higher in UM patients than in healthy controls [124]. An altered molecular composition of EVs has indeed been reported in several models of UM and in clinical trials. For example, miR-370, miR-210, miR-320a, miR-124, miR-107 and miR-486-5p were highly expressed in EVs from liver perfusate of UM patients with liver metastases, similar to the A375 melanoma cell line, but not in various other cancer cell lines [124]. Elsewhere, micro-RNA content, namely, miR-21, miR-34a, miR-126, of EVs isolated from vitreous humor of UM patients, was significantly elevated as compared to healthy controls [125]. UM also alters the protein profile of EVs, e.g., the content of heat shock proteins, members of integrin, and EGFR signaling was different in EVs from either MP41, MP46, MP92.1, Mel270, Mel285, OMM1.3 or OMM2.5 cells to that of control melanocytes [126,127]. In clinical studies, however, mostly inflammatory proteins, such as interferon-gamma, interleukin 2, 22 and 12 (p40), Pentraxin-3, TNFSF13B and TNFSF8 were significantly enriched in EVs from patients with metastatic UM [128]. The role of EV cargo in the mechanism of UM metastasis has also been shown [126]. UM EVs may contribute to metastatic niche preparation, and hepatocytes exposed to UM EVs induce migration of UM cells [126,127]. However, so far, a common feature in the vesicular signature of UM has not been proposed; thus, further studies are needed to assess the feasibility of an EV-based diagnostic tool for UM. 

As antineoplastic agents are capable of interfering with EV biology, it can be postulated that EVs may also be utilized to assess the efficacy of UM treatments. Vesicular effects of HDAC inhibitor in UM have not yet been studied in detail. In models of muscular dystrophy, Trichostatin A, an inhibitor of HDAC classes I and II, increased the amount of EVs and the level of miR-206 in EVs released by fibro-adipogenic progenitor cells, 10- and 20-fold, accordingly [129]. In FEMX-I metastatic melanoma, induction of CD133^+^ EV-release resulted from treatment with the HDAC6i Tubacin; however, other HDACi such as ACY-1215 or Trichostatin A caused a reduction in the number of EVs released [130]. Although direct evidence is yet to be provided in UM, these findings suggest that modulation of EV release or composition may not be a class-effect of HDAC inhibitors, which may limit prognostic value of EVs in UM treatment by HDAC inhibitors. Nevertheless, identifying favorable molecular changes induced by specific HDAC inhibitors in EVs may lead to the development of novel therapeutic systems.

## 9. Conclusions

A combined therapeutic approach—HDAC6i with chemotherapeutic agents—may be more beneficial and should be explored in detail as treatment options for UM/MUM. Promising results from Entinostat pembrolizumab gives us hope that it is worthwhile to pursue combinatorial treatment strategy in UM/MUM. Furthermore, cancer EVs need to be thoroughly investigated as either a vehicle for targeted drug delivery and/or as a therapeutic option for MUM. 

## Figures and Tables

**Table 1:** HDAC inhibitors in clinical trial for UM and advanced UM.

Drug	HDAC Class	Clinical Trial Identifier	Phase	Status
Entinostat + Pembrolizumab	Class I	NCT02697630	II	Ongoing
		NCT03765229	II	Recruiting
Vorinostat	Pan-HDAC	NCT00121225	II	Completed
		NCT01587352	II	Ongoing
		NCT03022565	-	Withdrawn
Valproic acid	Pan-HDAC	NCT02068586	II	Ongoing
Belinostat + Binimetinib	Pan-HDAC	NCT05170334	II	Recruiting

**Table 2:** HDAC6 inhibitors in clinical trials for various cancers.

Cancer Type	Drug Combination	Clinical Trial Identifier(s)	Phase	No. of Participants	Status	Outcome	Reference (PMID)
Biliary Tract Cancer (advanced)	KA2507	NCT04186156 EUCTR2019-001459-38-GB	Ib/II	N/A	Withdrawn	N/A	
Relapsed/Refractory Multiple Myeloma	ACY-1215 + Bortezomib/Dexamethasone	NCT01323751	I/II	120	Completed	N/A	
Lymphoid Malignancies, Lymphoma	ACY-1215	NCT02091063	I/II	24	Completed	Under review	
Non-Small Cell Lung Cancer	ACY-241 + Nivolumab	NCT02635061	Ib	17	Active, not recruiting	Ongoing	34552864
Solid Tumor, Adult	KA2507	NCT03008018	I	20	Completed	Published	33947698
Metastatic Breast Cancer, Breast Carcinoma	ACY-1215 + Nab-paclitaxel	NCT02632071	Ib	17	Completed	N/A	
Unresectable/Metastatic Cholangiocarcinoma	ACY-1215 + Gemcitabine/Cisplatin	NCT02856568	Ib	N/A	Withdrawn	N/A	
Relapsed/Refractory Multiple Myeloma	ACY-1215 + Pomalidomide/Dexamethasone	EUCTR2014-002338-29-IT EUCTR2014-002338-29-GR NCT01997840	Ib/II	103	Active, not recruiting	Ongoing	
Gynecological Cancer	ACY-1215 + Paclitaxel/Bevacizumab	NCT02661815	Ib	6	Terminated	Available	
Malignant Melanoma	ACY-241 + Nivolumab + Ipilimumab	NCT02935790	Ib	1	Completed	N/A	
Solid Tumor (advanced)	JBI-802	NCT05268666	I/II	126	Recruiting	N/A	

**Table 3:** Concentration of HDAC6 inhibitors used in studies.

Cancer Cell Line(s)	HDAC6 Inhibitor	Reported IC_50_ Value	Concentration Used	Reference (PMID)
HAP1-KO (HDAC6)	Tubathian A (8A)	1.9 nM	1, 10 and 50 µM	30694564
	Tubastatin A (Tub A)	15 nM	1, 10 and 50 µM	
	Tubacin (Tub)	4 nM	1 and 10 µM	
	Ricolinostat (ACY-1215)	4.7 nM	1 and 10 µM	
A375 HDAC6-KO DLD1 HDAC6-KO MDA-MB-231 HDAC6-KO TOV-21G HDAC6-KO	Ricolinostat (ACY-1215) Citarinostat (ACY-241)	4.7 nM	0.1–100 µM	31511426
		2.6 nM		
SMG5 and SMG6	Ricolinostat (ACY-1215)	4.7 nM	0–50 µM	33322608
Mel270, Mel285 and OMM2.5	Ricolinostat (ACY-1215)	4.7 nM	1–50 µM	35159049

## Data Availability

Not applicable.
